# Semi-quantitative Multispectral Optoacoustic Tomography (MSOT) for volumetric PK imaging of gastric emptying

**DOI:** 10.1016/j.pacs.2014.06.001

**Published:** 2014-06-27

**Authors:** Stefan Morscher, Wouter H.P. Driessen, Jing Claussen, Neal C. Burton

**Affiliations:** iThera Medical, GmbH, Munich, Germany

**Keywords:** MSOT, Multispectral Optoacoustic Tomography, ICG, Indocyanine Green, Multispectral Optoacoustic Tomography (MSOT), Photoacoustic imaging, Indocyanine Green, *In vivo* imaging, Pharmacokinetics, Gastric emptying

## Abstract

A common side effect of medication is gastrointestinal intolerance. Symptoms can include reduced appetite, diarrhea, constipation, GI inflammation, nausea and vomiting. Such effects often have a dramatic impact on compliance with a treatment regimen. Therefore, characterization of GI tolerance is an important step when establishing a novel therapeutic approach.

In this study, Multispectral Optoacoustic Tomography (MSOT) is used to monitor gastrointestinal motility by *in vivo* whole body imaging in mice. MSOT combines high spatial and temporal resolution based on ultrasound detection with strong optical contrast in the near infrared. Animals were given Indocyanine Green (ICG) by oral gavage and imaged by MSOT to observe the fate of ICG in the gastrointestinal tract. Exponential decay of ICG signal was observed in the stomach in good correlation with *ex vivo* validation. We discuss how kinetic imaging in MSOT allows visualization of parameters unavailable to other imaging methods, both in 2D and 3D.

## Graphical abstract

**Figure:**
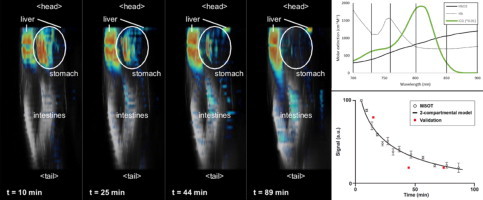

## 1 Introduction

Lifestyle and medication can have a significant impact on gastric emptying. For example, alcohol, tobacco and nicotine reduce gastric motility, whereas beta-adrenergic receptor antagonists and erythromycin have prokinetic properties [1]. These effects can influence the exposure to medication by increasing or decreasing bioavailability and can impact compliance to the therapeutic regimen. A variety of methods have been employed to interrogate the gastrointestinal (GI) system to characterize the effect of xenobiotics. Some of them involve invasive surgeries where smooth muscle electrical activity is measured by attaching electrodes and miniaturized strain-gauge transducers directly to muscle tissue [2], [3], [4]. Similarly, sonomicrometry can be employed by attaching multiple piezoelectric transducers directly to tissue, and characterizing motion by determining the time-interval between the detection of acoustic waves at the different transducers [5]. Muscle activity can also be measured with *ex vivo* organotypic muscle tissue cultures, allowing tight control over experimental conditions [6]. Terminal approaches can be used to measure stomach contents *ex vivo* [7], [8], or determine the mobilization of dyes fed to the animal throughout the gut *via* histology [9], [10].

Non-invasive *in vivo* methods have also been developed to probe gastric function. For example, glass beads can be fed to mice, and the colonic expulsion time can be taken as a metric of whole gut transit time [11]. Similarly, dyes can be used to assess the gut latency by observing the first appearance of the agent in the stool [12]. Other metrics such as food intake, fecal pellet weight and percentage of animals exhibiting diarrhea can also be used as gross indicators of gastric intolerance.

*In vivo* imaging has also been used to measure gastric motility. For instance, radiopaque or radiolabeled substances can be administered, and the distribution of the agent can be visualized through X-ray photography [13] or through gamma scintigraphy [14]. Alternatively, fluorescence reflectance imaging has been used to show the distribution of administered agents throughout the gut [15]. However, these imaging approaches have been limited to dorsal views of the animal where the distinction between stomach, liver and intestines is challenging due to photon scattering along the detection path. High resolution imaging of optical contrast in biological tissue requires an approach to overcome optical tissue scattering. Acoustic waves are less subject to scattering, indicating that a hybrid imaging modality can obtain higher resolution in whole body imaging. Acousto-optic imaging [16], [17] uses spatially restrained ultrasound pulses to modulate light pulses that can in turn be detected with a photodetector, hence allowing one to obtain a more accurate measure of tissue absorption. However, the portion of light being modulated and thereafter detected is comparably small, and is still affected by absorption in tissue, limiting sensitivity of the modality. The presented work uses the photoacoustic effect to generate sound waves, which are far less subject to scattering in tissue. While some approaches exist that exploit this effect using microwave excitation in the near-field [18], [19], [20] or amplitude modulated light sources to employ optoacoustic imaging in the frequency domain [21], [22], time domain optoacoustic imaging is the most commonly used implementation. With existing implementations in photoacoustic microscopy [23], [24], [25], [26] and high-resolution tomography using frequencies above 10 MHz [27], [28], [29], this paper applies lower detection frequencies for whole body optoacoustic tomography [30], [31], [32].

Multispectral Optoacoustic Tomography (MSOT) [33], [34] is an *in vivo* optical imaging modality that, in the presented study, enables non-invasive assessment of gastric emptying with high spatial and temporal resolution. The animal is illuminated with short, near infrared (NIR) light pulses at different wavelengths generated by a tunable laser. This light is absorbed by tissue-intrinsic chromophores such as hemoglobin or melanin or administered exogenous contrast agents with distinct absorption spectra in the near infrared, such as Indocyanine Green (ICG). Importantly, MSOT does not rely on emitted fluorescence like other optical imaging modalities, but mere absorption of light is enough to give rise to the photoacoustic effect. Following absorption, part of the absorbed energy is converted to heat, causing a transient thermoelastic expansion that generates an acoustic wave. This acoustic wave can be detected by an array of ultrasound transducers to produce an optoacoustic image representing an acoustic pressure map. In the chosen implementation, such a map depicts a cross-section of a mouse and, using assumptions on light fluence, can be converted to a map of optical absorption. This allows discrimination between signal intensities in multiple organs; for example, the stomach and liver in the current study. Image acquisition at multiple wavelengths and subsequent spectral analysis [35] allows the determination of the spatial distribution of NIR-absorbing agents in distinction to tissue-intrinsic contrast in many scenarios of applied research [34], [36], [37], [38], [39], [40]. An optoacoustic pressure map can be generated within microseconds based on simultaneous detection of signals from all elements of the transducer array, allowing a high temporal resolution only limited by the feasible laser repetition rate. Unlike conventional optical imaging, optoacoustic imaging maintains its high spatial resolution also in deep tissue, since acoustic waves can traverse tissue with almost undiminished quality, whereas photons are subject to a comparatively high amount of scattering [41], [42].

MSOT has further been used in this regard for imaging of dynamic contrast enhancement with extrinsic contrast agents in the brain [36], [37] and various metabolic organs [43], [44]. To take this concept further, the presented study uses dynamic contrast enhanced imaging for investigation of stomach emptying.

## 2 Methods

### 2.1 Animals

Eight week old nude Foxn1 female mice were used in compliance with the Helmholtz Zentrum München Animal Care and Use committee. The mice were fasted for a brief period before the experiment to allow comparable results. During the experiment the animals were under isoflurane anesthesia (1.8% at 0.8 l/min).

### 2.2 Imaging agents

**Fig. 1:**
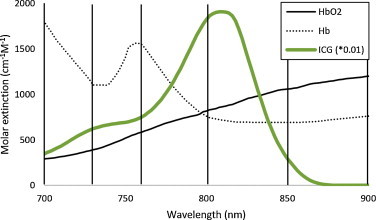
Near infrared absorption spectra of hemoglobin as the dominant background absorber (de-oxygenated and oxygenated) and ICG [45], with highlighted optoacoustic imaging wavelengths (700 nm, 730 nm, 760 nm, 800 nm, 850 nm, 900 nm).

Indocyanine Green (ICG) (Pulsion Medical Systems, Germany) was selected due to its well-studied fluorescence and optoacoustic properties [45]. Its absorption profile in plasma is shown in Fig. 1 (at 6.5 μM). It is an FDA-approved, water-soluble, inert anionic tricarbocyanine dye. For gavage studies, 10 nmol of ICG was mixed into a 200 μl solution containing 1% Cremophor (v/v), 25% glycerol (v/v) in water, and the volume was injected into the animal *via* a feeding tube immediately prior to MSOT imaging. This vehicle was found to be well tolerated by the animals in earlier studies [46].

### 2.3 Experimental MSOT imaging system

**Fig. 2:**
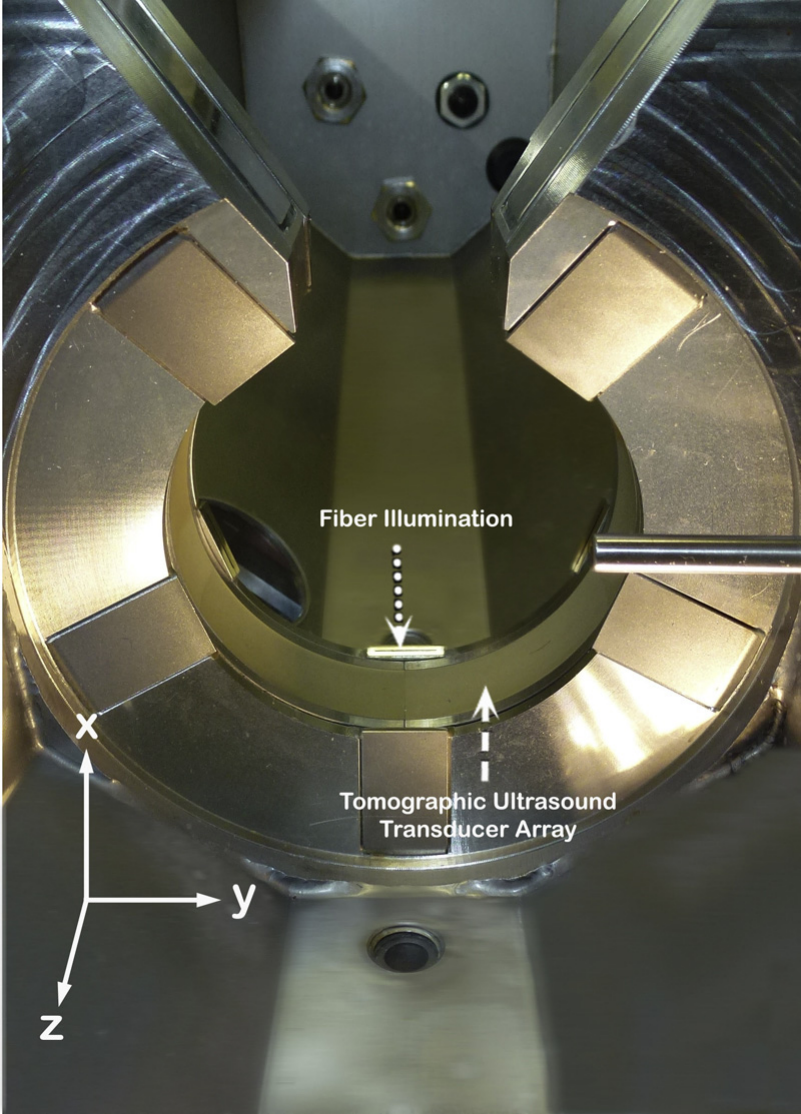
Illumination and detection geometry in the imaging chamber. The animal is translated along the *z*-axis in order to enable acquisition of multiple transverse cross sections in the *x*–*y* plane.

The experimental MSOT imaging setup used for most of this study was engineered at the Institute of Biological and Medical Imaging (IBMI) at Helmholtz Zentrum München and will be referred to as “experimental imaging system” in the rest of this paper. Earlier versions of this system have been described elsewhere in detail [47], [48]. A tunable optical parametric oscillator (OPO) pumped by an Nd:YAG laser (Opotek Inc., Carlsbad, CA) provides excitation pulses with a duration of 9 ns at wavelengths from 680 nm to 980 nm at a repetition rate of 10 Hz. A light strip of about 8 mm width on the mouse is evenly illuminated from 10 arms of a fiber bundle arranged at an angle of 13 degrees to the imaging plane (see Fig. 2). The laser delivers a peak energy of 100 mJ at 730 nm, which is distributed across 6 cm^2^ on the mouse surface, resulting in a radiant exposure of 18.3 mJ/cm^2^ which is well below the maximum permissible exposure (MPE) in humans as defined in EN60825-1. In order to correct the acquired multispectral data, the laser's energy profile was measured at the wavelengths used for the acquisition of MSOT data. A cylindrically focused 128 element ultrasound transducer array at a center frequency of 5 MHz covers an angle of 270 degrees around the sample to create cross-sectional images. The photoacoustic signals in the μV range captured by all transducer elements are digitized simultaneously using specialized acquisition electronics (Falkenstein Microsysteme GmbH, Taufkirchen, Germany) at a sampling rate of 40 megasamples/s. Mice are submerged in a water tank in a horizontal position in a holder and are wrapped in a thin polyethylene membrane to prohibit direct contact between water and mouse but still allow for acoustic coupling. Anesthesia and oxygen are supplied through a breathing mask. The mouse and holder can be translated using a linear stage (IAI Industrieroboter GmbH, Schwalbach, Germany) to enable imaging of multiple transverse slices. In order to create an image at one wavelength, signals from 20 subsequent excitation pulses were averaged in order to compensate for laser pulse fluctuations and animal motion as well as to improve signal-to-noise ratio (SNR).

In the second part of the study, imaging was performed using an MSOT inVision 256-TF small animal scanner [48] (iThera Medical GmbH, Munich, Germany) that will from here onwards be referenced as “MSOT inVision system”. It is an improved and commercially available implementation of the imaging system detailed above, featuring a 256 element transducer array at the same center frequency, but using a reduced radius for the active surface to create a toroidal focusing that allows for an increased field of view in the imaging plane. It also uses a specifically developed, proprietary laser system (InnoLas Laser GmbH, Krailling, Germany) of equal energetic characteristics and pulse width, but in contrast to the prototype detailed above allows fast wavelength tuning in between laser pulses. It also features an integrated laser pulse energy correction that enables the correction of each laser pulse with its measured energy, inherently compensating for the laser's wavelength-dependent energy profile. This reduces the need for averaging and thus greatly enhances imaging speed (one multispectral image in 7 s using 10 averages and 7 wavelengths), allowing for acquisition of multiple slices per time point to capture kinetic processes in volumetric data sets as demonstrated in the study.

The vertical lines in Fig. 1 depict the excitation wavelengths at which *in vivo* MSOT measurements are made, representing wavelengths with distinctive absorption patterns relative to the other background chromophores depicted in the graph.

### 2.4 Image reconstruction and spectral unmixing

**Fig. 3:**
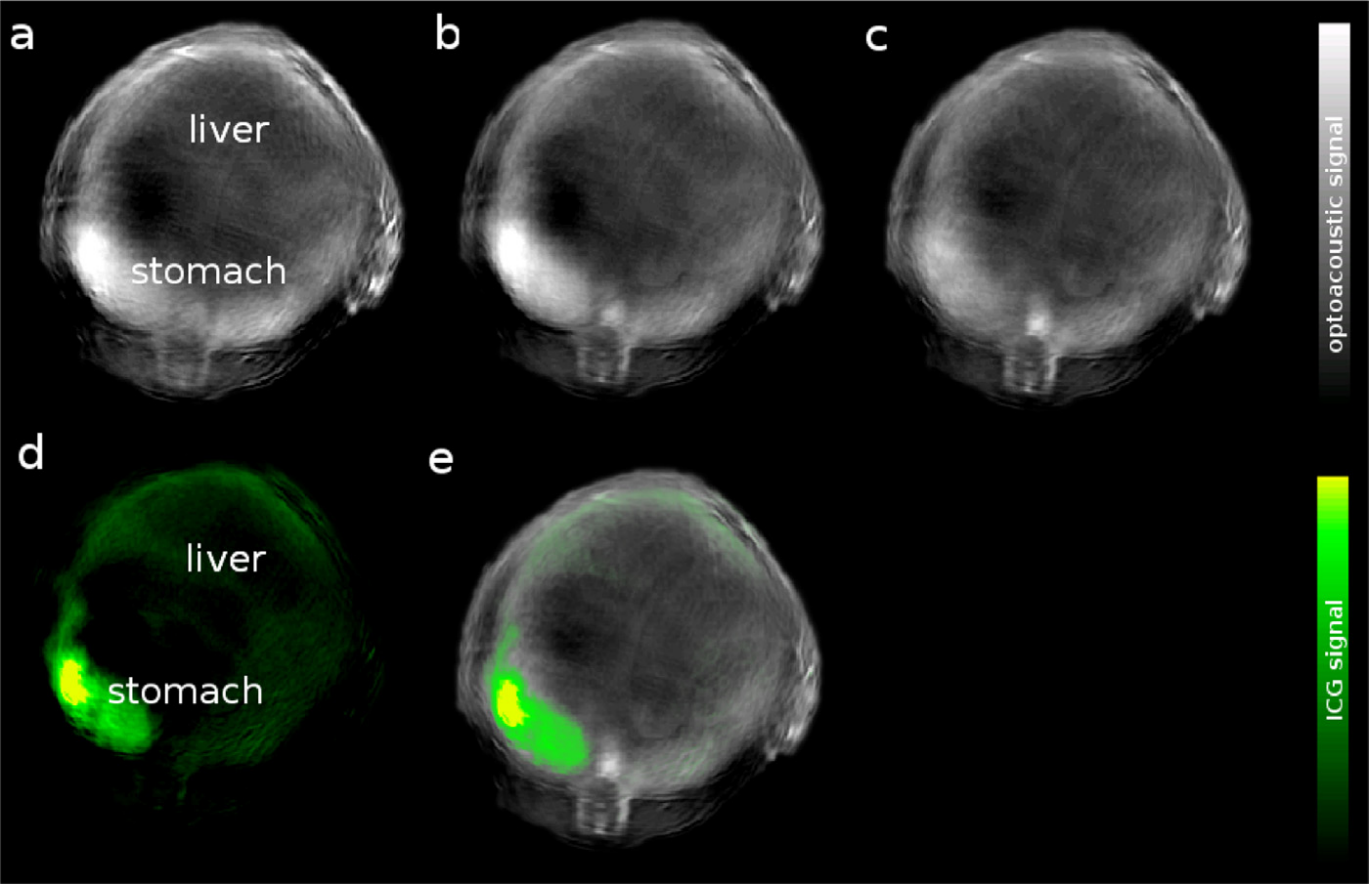
Combined visualization of anatomical and molecular images: Optoacoustic images are shown 5 min after oral gavage of ICG. (a–c) Single-wavelength images at *λ* = (a) 700 nm, (b) 800 nm, (c) 900 nm; (d) biodistribution of ICG obtained through multispectral unmixing (e) overlay of single-wavelength background image (*λ* = 900 nm) representing anatomical structures in grayscale with probe biodistribution information from the ICG component in color. For an annotated cryoslice image of a similar mouse showing reference anatomy see Fig. 4b.

**Fig. 4:**
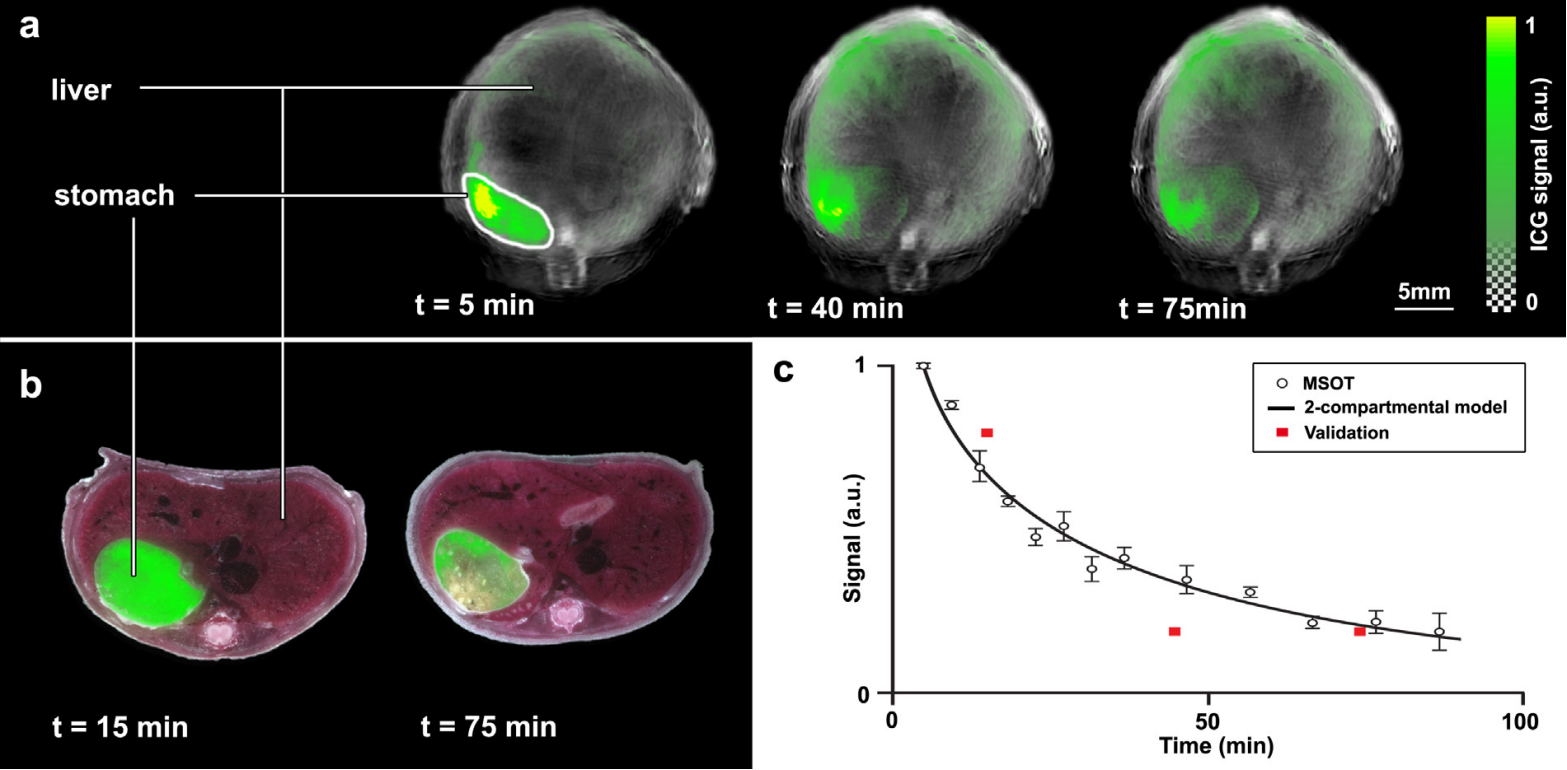
MSOT imaging of gastric emptying. Panel (a) shows the ICG signal distribution at multiple time points after oral gavage of an ICG-containing solution. MSOT images show a single-wavelength optoacoustic image (grayscale, 900 nm) as an anatomical reference with an overlay of multispectrally resolved ICG signal (green). Panel (b) shows *ex vivo* sectioning of animals sacrificed at 15 and 75 min post ICG administration, with an RGB image showing reference anatomy and location of the fluorescent ICG shown in green. Panel (c) shows the quantification of MSOT signals from mice (*n* = 3) (open circles), with modeled data shown as a black line. Red squares indicate fluorescence measured *ex vivo* in mice (*n* = 1).

**Fig. 5:**
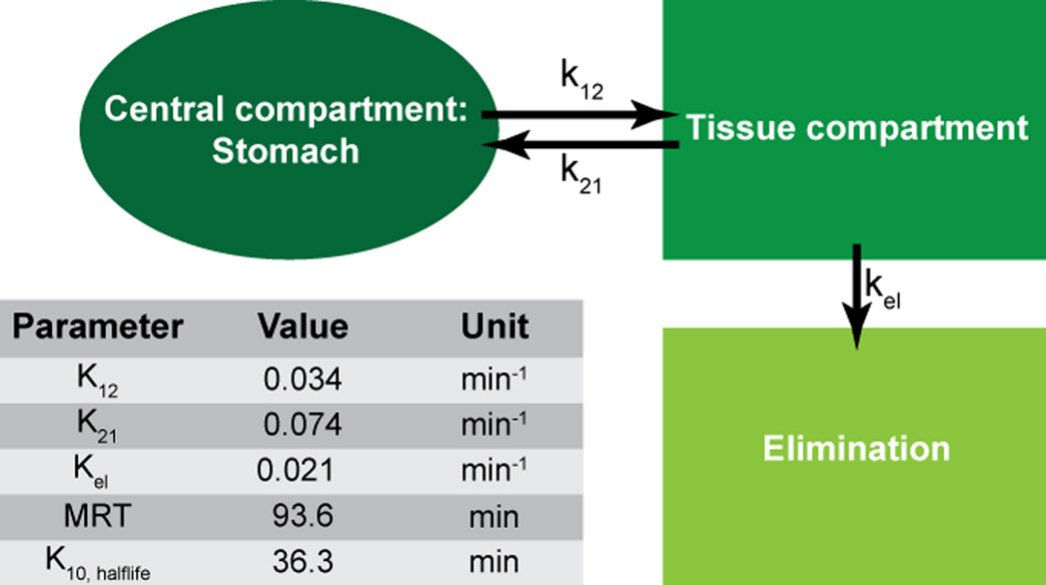
Pharmacokinetic model employed on the MSOT-derived concentration–time profiles and parameters describing the modeled curve shown in Fig. 4c.

Images in Fig. 3, Fig. 4 were reconstructed using a standard backprojection algorithm [49], and three-dimensional images in Fig. 5 were reconstructed using the interpolated model-matrix inversion [50]. Both were applied from within the ViewMSOT software suite supplied with the iThera Medical system. After image reconstruction, linear spectral unmixing was applied to detect and separate signals from photo-absorbing tissue elements, such as hemoglobin, or ICG [35], [51]. For each pixel in the image, the method fits the total measured optoacoustic spectrum to the known absorption spectra of oxy- and deoxyhemoglobin and that of the agent to be detected. This produces individual component images, each visualizing the bio-distribution of the respective absorber. In the presented study this process is specifically employed to be able to separate changes in the ICG signal from changes produced by hemoglobin, *e.g.* a difference in perfusion.

### 2.5 Image quantification and presentation

**Fig. 6:**
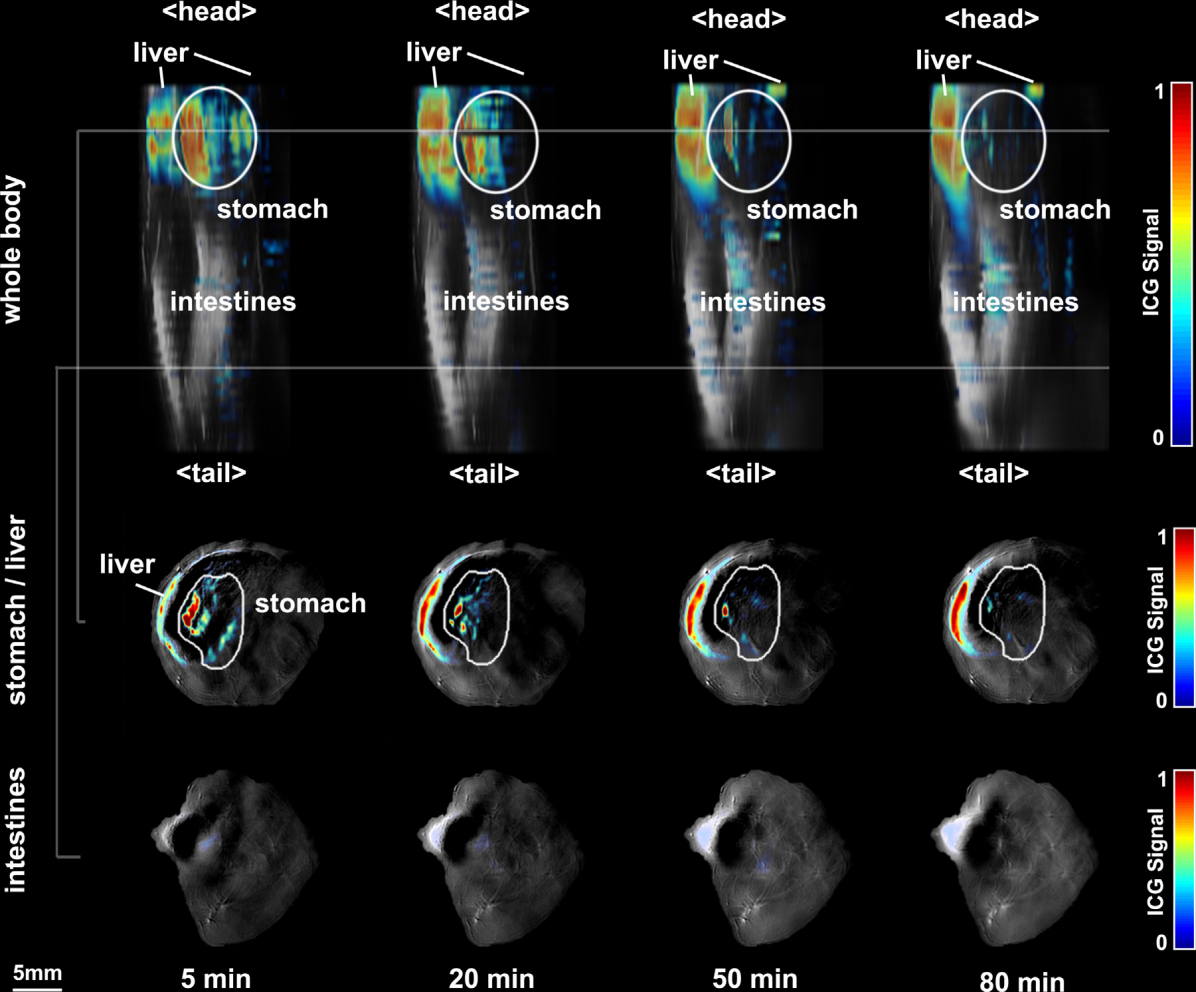
Three-dimensional MSOT kinetics: Volumetric representation of anatomical information in grayscale combined with molecular information overlaid in color at different time points (*t* = 5 min, *t* = 20 min, *t* = 50 min, *t* = 80 min), both in a 3-D rendering (top row) and in two exemplary cross-sections in stomach and intestines (bottom two rows). ICG clears from the stomach and accumulates in the intestines and the liver.

In order to also visualize deep structures such as the stomach correctly in 3D, the images in Fig. 6 have been corrected with an approximate light fluence model based on the finite elements approach applied to generate the forward model in [52], [53]. It was based on a simple, cylindrical FEM mesh adapted to the size of the mouse and the imaged volume, while assuming homogeneous distribution of scattering and absorption (model parameters: background tissue oxygenation = 70%, $\mu_{\text{a}}^{800\;\text{nm}}=0.3{\text{ cm}}^{-1}$, ${\mu^{\prime }}_{\text{s}}=10{\text{ cm}}^{-1}$) within the subject. Based on its simplicity, this model does not allow quantification of signal but merely represents a mode to adequately visualize deep seated signals.

Data in Fig. 3, Fig. 4 has not been corrected for locally varying light fluence, hence signal intensities will be varying with depth. In awareness of this fact, regions of interests have been selected at similar size (∼25 mm^2^) and depth (2–6 mm). The signal amplitudes in Fig. 4c were calculated from the mean of the selected area in the ICG component image and were individually normalized to the peak intensity of each animal for the modeling.

### 2.6 Pharmacokinetic modeling

Pharmacokinetic modeling of the concentration–time curves was performed using a two-compartmental open body model in WinNonLin (Certara L.P., St. Louis, MO, USA) [54]. This pharmacokinetic model was chosen based on the evaluation of the Goodness-of-Fit (correlation between observed and predicted values; residual plots). As in all compartmental models, homogenous distribution of compound within each compartment and a non-changing volume of each compartment is assumed for the modeling.

### 2.7 Three-dimensional visualization

The three-dimensional renderings presented in Fig. 5 were created outside the ViewMSOT software supplied with the iThera Medical system by using the shear warp rendering algorithm [55] from the viewer_3d toolkit (version 11) available through MATLAB Central File Exchange [56]. Logarithmic alpha mapping was applied in order to be able to visualize signals deep inside the animal.

### 2.8 Cryosectioning with planar fluorescence imaging

For whole-body, *ex vivo*, cross-sectional fluorescence imaging of probe biodistribution, a Leica cryostat (CM 1950, Leica Microsystems, GmbH, Wetzlar, Germany) was retrofitted with a fluorescence imaging system [57] with excitation at 740 nm and emission captured with a 780 nm long pass filter. The exposure time was adjusted dynamically with an upper limit of 7s to optimize the dynamic range and stored with the image. The evaluated images were divided with their respective exposure time in order to put them on a comparable scale. Prior to measurements a test target was imaged in order to calibrate measurements and identify potential differences in the illumination between samples.

The samples were cut at equidistant intervals of 500 μm throughout the region of the stomach. One slice was selected from each sample based on visual similarity with the structures displayed in the MSOT images as well as a representative amount of ICG that matched the adjacent slices.

## 3 Results

### 3.1 Separation from background absorbers

In order to evaluate the biodistribution of ICG from MSOT images, spectral unmixing needs to be performed to separate the agent signal from the background signals. The first row of images in Fig. 3 shows exemplary cross-sectional optoacoustic images at three of the measured wavelengths, acquired on the experimental imaging system. Imaging for all mice shown in this study was performed in an area of the animal that shows both the stomach and the liver. The second row shows the component image for ICG resulting from spectral unmixing, showing the distinct contributions of the individual absorber separated from the strong hemoglobin background visible in the first row. In order to better visualize the bio-distribution of ICG, the respective component is overlaid with a green color map onto a non-specific single-wavelength image in grayscale to combine probe and background tissue information.

### 3.2 PK imaging of stomach emptying

In order to observe the kinetic process of stomach emptying, imaging was performed continuously to produce a time series of images with the experimental imaging system. The first row of Fig. 4 shows three images at different time points after the oral gavage of ICG. It clearly shows a decrease of specific ICG signal in the stomach area and at the same time an increase of ICG signal in the liver area following the known hepatobiliary clearance pathways of ICG after systemic absorption. *Ex vivo* fluorescence cryoslice data from different animals undergoing the same procedure show the same behavior, which is depicted in the second row of Fig. 4. Most interestingly, the signal decay in the stomach through clearance can be observed by defining a region of interest in the stomach and following the mean signal intensity of the unmixed ICG component in the selected area. The graph in Fig. 4c shows the respective data over time from animals undergoing the same procedure (*n* = 3; open circles). This data can be fitted to a two-compartmental model (Fig. 5). *Ex vivo* cryoslice validation confirms the results observed by MSOT imaging at 3 time points (Fig. 4; red squares).

The MSOT-derived concentration–time profiles were modeled to a two-compartmental open body model (Fig. 5) to account for the biphasic gastric emptying behavior observed. The rate constants describing the modeled PK curve are found in Fig. 5. ICG displays a mean residence time (MRT) of 93.6 min in the stomach and an elimination half-life of 36.3 min.

### 3.3 Volumetric PK imaging

Using the MSOT inVision imaging system, it was possible to visualize the same process in multiple cross-sectional slices, allowing the volumetric visualization of gastric emptying with a temporal resolution of 5 min. Selected time points are shown in Fig. 6 with a three-dimensional rendering (first line) and exemplary cross-sectional images from two locations along the *z*-axis representing the stomach/liver area and an intestinal area (gray lines). All time points are combined in a video (see Video 1). Equivalent to the observations made In Fig. 4, signal from ICG in the stomach area is decreasing with time, as ICG is cleared from the stomach toward the intestines. As the stomach empties into the intestines, some of the ICG is likely absorbed in the proximal duodenum, accounting for the increased liver signal following hepatobiliary excretion.

## 4 Discussion

As MSOT imaging is non-invasive, it is possible to repeatedly image individual animals, enabling longitudinal imaging, *e.g.* to characterize a baseline rate of gastric emptying against which comparisons can be made following treatment. This approach can potentially minimize the impact of biological variability between mice, and allows the use of fewer animals to make a statistically significant observation. Indeed, since it is not necessary to sacrifice the animal to obtain a measurement, it is also possible that the animals may be permitted to recover from drug treatment before being used again, depending on the nature of the toxicant or pharmaceutical being tested. This could also aid in the characterization of chronic exposure to xenobiotics and related effects on gastric emptying and thus provide insights for pre-clinical research in characterization of novel pharmaceuticals. However, the presented study cannot be easily translated to clinical settings due to limitations of penetration depth.

The non-invasive aspect of MSOT-based gastric emptying determination is also important in terms of allowing the investigator access to biological processes in the intact animal. Organotypic cultures or *ex vivo* observations can sometimes be subject to artifacts related to euthanasia, or the necessity of making conclusions on tissue outside of its native environment [58]. Through the use of *in vivo* imaging, one can make a direct conclusion on gastric motility without disrupting the gastrointestinal system.

In addition, the acquisition of single-wavelength data at 10 images per second frame rate, or multispectral data at up to 2 images per second, yields sample-rich data sets. In comparison to *ex vivo* studies, pharmacological modeling with MSOT requires far fewer animals. Traditionally, kinetic studies are performed by sacrificing many animals at multiple time points, giving rise to study cohorts with dozens of animals per treatment group. Instead, as in this study, a full pharmacological profile can be determined using MSOT with a significantly smaller number of mice, driving down the financial investment of the study, as well as the time required to complete it.

For the determination of gastric emptying *in vivo*, optoacoustic imaging has advantages over traditional optical imaging. First, as acoustic waves scatter far less compared to photons, it is possible with MSOT imaging to maintain resolution at depth. In contrast, optical imaging detects photons which have propagated through and have been scattered by tissue [52]. Without a mechanism to model the complex scattering of light, it is impossible to precisely determine the origin of optical signals at depth. Hence, spatial resolution of optical imaging greatly suffers in determining dynamics of the gut. In addition, the tomographic approach to optoacoustic imaging presented in this manuscript demonstrates the utility of cross-sectional *versus* projection-based planar imaging in discriminating various parts of the gastrointestinal system from adjacent organs, such as the stomach and liver. In projection based approaches, it can be hard to determine from a single view angle whether a superficial weak signal or a deeper strong signal is being detected. Therefore, one must rotate the animal repeatedly and acquire data from multiple view angles in order to increase confidence in signal localization, thereby increasing the effort and time required to conduct an experiment. With MSOT imaging, this is not necessary since the cross-sectional view provides a clear visualization of the spatial distribution of injected agents, while with the recent technological advances whole-body imaging by translation of the animal is feasible while maintaining high temporal resolution. Fast multispectral imaging thus allows the sequential acquisition of volumetric datasets to enable complex three-dimensional representation of biodistribution of agents. While the presented horizontally rendered view of the animal is very similar to what is known from projection-based planar optical imaging approaches, the additional cross-sectional images provide evidence of the actual spatial distribution of the agent in the animal. Apart from the inherent spatial and temporal resolution, the combination of tissue and probe information can be very useful in various fields to study biodistribution and kinetic processes in mice. The additional feature of probe multiplexing [40] by combining labels with different absorption peaks paves the road for even more advanced scenarios, such as the simultaneous injection of a functionalized agent and a control in the same animal or, in the case of the described application, simultaneously determining gastric emptying rates of liquids and solids.

While MSOT imaging can also provide very sensitive detection of signal in deep tissues, absolute quantification of signals remains still the most challenging aspect of optoacoustic imaging based on the need to accurately model light flux in deep tissues. It must be noted that in the presented study only the three-dimensional dataset shown in Fig. 6 was corrected using an approximate light fluence model for visualization of signals in deeper regions. The graph in Fig. 4 was created from regions that were intentionally selected with similar depth and size and therefore under the assumption of equal illumination conditions. While this holds for the presented imaging scenario for comparisons between animals in a similar imaging setting, this certainly poses a challenge for other applications that require comparisons between organs with very different optical parameters within a single animal.

In summary, MSOT offers important new features in small animal gastrointestinal imaging. It was demonstrated that anatomical and physiological parameters were visualized in high spatial and temporal resolution through gut cross-sections and volumes. In this study, non-targeted ICG was used to measure gastric emptying, but it would similarly be possible to conduct such an experiment with a molecular probe to detect, for example, inflammatory epitopes in the gut. This imaging ability may bring a new dimension in small animal GI research by allowing imaging of physiological and possibly molecularly targeted signals with high resolution; a capacity not available with optical or nuclear imaging techniques. We expect that the combination of the imaging performance exhibited by small animal MSOT together with new molecular probes will enable new insights into gastrointestinal function and disease.

As a modality, however, optoacoustic imaging is not limited only to the preclinical setting but has shown very promising perspectives in many scenarios in a clinical setting such as mammography [59], superficial imaging using handheld detectors [60], [61], [62] and intravascular as well as endoscopic imaging [63], [64], [65].

## Conflict of interest

All authors are employees of iThera Medical GmbH.

## Appendix A Supplementary data

Video 1 Biodistribution of ICG over time in 3D.
